# Floral to green: mating switches moth olfactory coding and preference

**DOI:** 10.1098/rspb.2011.2710

**Published:** 2012-02-08

**Authors:** Ahmed M. Saveer, Sophie H. Kromann, Göran Birgersson, Marie Bengtsson, Tobias Lindblom, Anna Balkenius, Bill S. Hansson, Peter Witzgall, Paul G. Becher, Rickard Ignell

**Affiliations:** 1 Chemical Ecology Group, Swedish University of Agricultural Sciences, 230 53 Alnarp, Sweden; 2 Department of Evolutionary Neuroethology, Max Planck Institute for Chemical Ecology, Hans-Knoell-Strasse 8, 07745 Jena, Germany

**Keywords:** olfaction, mating, modulation, host finding, herbivore, *Spodoptera littoralis*

## Abstract

Mating induces profound physiological changes in a wide range of insects, leading to behavioural adjustments to match the internal state of the animal. Here, we show for the first time, to our knowledge, that a noctuid moth switches its olfactory response from food to egg-laying cues following mating. Unmated females of the cotton leafworm (*Spodoptera littoralis*) are strongly attracted to lilac flowers (*Syringa vulgaris*). After mating, attraction to floral odour is abolished and the females fly instead to green-leaf odour of the larval host plant cotton, *Gossypium hirsutum*. This behavioural switch is owing to a marked change in the olfactory representation of floral and green odours in the primary olfactory centre, the antennal lobe (AL). Calcium imaging, using authentic and synthetic odours, shows that the ensemble of AL glomeruli dedicated to either lilac or cotton odour is selectively up- and downregulated in response to mating. A clear-cut behavioural modulation as a function of mating is a useful substrate for studies of the neural mechanisms underlying behavioural decisions. Modulation of odour-driven behaviour through concerted regulation of odour maps contributes to our understanding of state-dependent choice and host shifts in insect herbivores.

## 1. Introduction

Insects largely rely on their sense of smell to find mates, food and larval host plants for oviposition. They detect and discriminate between multi-component odours with astounding sensitivity and specificity against a noisy background, and they reliably track these odours during upwind flight [1–3]. Plasticity adds yet another dimension to the sense of smell—the olfactory system is under modulation to match the internal physiological state of the animal with external sensory stimuli [4].

Mating induces profound physiological changes, especially in females, that necessitate changes in foraging and reproductive behaviour [4]. The response of female mosquitoes to host and oviposition cues changes following feeding and mating [5] and, mated Mediterranean fruit-flies become attracted to host fruit instead of male pheromone [6]. In *Drosophila melanogaster* females, mating leads to significant behavioural and physiological changes, including decreased receptivity, enhanced egg production and oviposition, and a dietary switch towards protein-rich food [7–9], while it is still unclear whether mating modulates olfaction in *D. melanogaster*. A post-mating modulation of the olfactory system, which accounts for behavioural changes, has been studied only in males of the noctuid moth *Agrotis ipsilon*, which undergo a transient response inhibition to female sex pheromone [10].

We here show that mating modifies the olfactory physiology of cotton leafworm females *Spodoptera littoralis* (Lepidoptera, Noctuidae), leading to a shift in olfactory preference that matches their reproductive state.

## 2. Materials and methods

### (a) Insects

Cotton leafworm (*Spodoptera littoralis)* was obtained from cotton fields (El-Shatby, Egypt) in 2010 and reared on a standard semi-artificial agar-based diet. All insects were maintained under a 16 L : 8 D photoperiod, at 23 ± 1°C and 50–60% relative humidity (RH), adults had access to water and sugar solution. Two to 3 days-old unmated and 24 to 27 h post-mated female moths were used in all bioassays.

### (b) Plant material

Cotton seedlings (*Gossypium hirsutum* L., cv. Delta Pineland 90) were grown singly at 25 ± 5°C and at 70 ± 5% RH, under daylight and an artificial light source (400 W). Cotton plants used in behavioural experiments had 8–10 fully developed true leaves. Freshly cut lilac flowers (*Syringa vulgaris* L.) were field-collected (Alnarp, Sweden) during June and July.

### (c) Chemicals

Synthetic compounds were diluted in redistilled *n*-hexane. Compounds that gave a response in calcium imaging recordings (see §2*h*) were all greater than 98 per cent pure: nonanal (CAS number 124-19-6; Fluka), decanal (112-31-2; Sigma), (*Z*)-3-hexenyl acetate (3681-71-8; Aldrich), benzyl alcohol (100-51-6; Aldrich), benzaldehyde (100-52-7; Aldrich), phenylacetaldehyde (122-78-1; Acros), benzyl methyl ether (538-86-3; Aldrich), acetophenone (98-86-2; Acros), (*R*)-(−)- and (*S*)-(+)-linalool (126-90-9 and 126-91-0, respectively; Firmenich), lilac alcohols and aldehydes, each of which comprises a blend of eight stereoisomers [11] (Stefan Dötterl, Bayreuth).

### (d) Plant headspace collections

Plant odour was collected using a dynamic headspace apparatus. A freshly cut cluster of lilac flowers (without green leaves) was enclosed in a 2 l glass jar, which was closed with a grounded glass fitting. The cut end of the branch was placed in a 10 ml glass vial with water. A charcoal-filtered continuous air stream (150 ml air min^−1^) was drawn by a diaphragm vacuum pump (KNF Neuberger, Freiburg, Germany) over the flowers, from the bottom to the top of the jar, onto an air filter [12]. Volatiles were collected during 8 h at 3–4 lux at 22 ± 1°C.

A cotton plant was placed inside a 5 l glass cylinder. The bottom of the stem was held tightly in an orifice (*ca* 1 cm diameter), shaped by two adjacent glass panes that separated the green part of the cotton plant from the pot with soil holding the roots. A charcoal-filtered continuous air stream (350 ml min^−1^) was blown into the glass cylinder by a vacuum pump. At the top of the cylinder, air was drawn through two outlets, situated opposite to each other, onto two air filters (150 ml min^−1^, each). The excess air left the jar via the opening around the stem, avoiding the entry of unfiltered air. Volatiles were collected during 24 h (12 L : 12 D) at 22 ± 1°C.

Air filters were made of glass tubes (4 × 40 mm), holding 50 mg Super Q adsorbent (80/100 mesh, Altech, Deerfield, IL, USA) between glass wool plugs. The filters were rinsed with 2 ml re-distilled ethanol and *n*-hexane (LabScan, Malmö, Sweden) before use. Adsorbed volatiles were desorbed by eluting filters with 500 µl re-distilled *n*-hexane. Headspace was collected during a total of 960 h from lilac flowers and during 1266 h from cotton foliage. These odour collections were pooled and condensed under a stream of nitrogen to contain 10 min equivalents µl^−1^, for chemical analysis, electrophysiological recordings and calcium imaging. For quantification, 1 µg of heptyl acetate (99.8% chemical purity; Aldrich) was added as an internal standard. Samples were stored in sealed glass capillary tubes at −20°C.

### (e) Chemical analysis

Plant volatile collections were analysed on a coupled gas chromatography–mass spectrometer (GC–MS; 6890 GC and 5975 MS; Agilent Technologies, Palo Alto, CA, USA), operated in the electron impact ionization mode at 70 eV. The GC was equipped with fused silica capillary columns (30 m × 0.25 mm, d.f. = 0.25 μm), DB-wax (J&W Scientific, Folsom, CA, USA) or HP-5MS (Agilent Technologies). Helium was used as the mobile phase at an average linear flow rate of 35 cm s^−1^. Two microlitres of each sample were injected (splitless mode, 30 s, injector temperature 225°C). The GC oven temperature for both columns was programmed from 30°C (3 min hold) at 8°C min^−1^ to 225°C (5 min hold). Separation and identification of the linalool enantiomers was performed on a fused silica capillary column (30 m × 0.25 mm) coated with HP-chiral 20B (d.f. = 0.25 µm; Agilent). Two microlitres were injected manually, splitless, during 6 s at 225°C, to enable sharp injections and narrow, separated peaks of the enantiomers. The GC was programmed from 80°C (2 min hold) at 10°C min^−1^ to 110°C, and was held isothermal for 3 min, while the linalool enantiomers eluted. Compounds were identified according to their retention times (Kovat's indices) and mass spectra, in comparison with a NIST library (Agilent) and authentic standards, on two columns.

### (f) Electrophysiology

Antennal responses of unmated and mated *S. littoralis* females to volatile collections from lilac flowers and cotton foliage and to synthetic compounds were studied by electroantennography (EAG) and combined GC and electroantennographic detection (GC–EAD), using an EAG apparatus (IDAC-2; Syntech, Kirchzarten, Germany) and an Agilent 6890 GC.

For GC–EAD recordings, GC columns and the temperature programmes were the same as for the GC–MS analysis. Hydrogen was used as the mobile phase at an average linear flow of 45 cm s^−1^. At the GC effluent, 4 psi of nitrogen was added and split 1 : 1 in a Gerstel 3D/2 low dead volume four-way-cross (Gerstel, Mülheim, Germany) between the flame ionization detector and the EAD. The GC effluent capillary for the EAD passed through a Gerstel ODP-3 transfer line, that tracked GC oven temperature, into a glass tube (30 cm × 8 mm), where it was mixed with charcoal-filtered, humidified air (18–20°C, 50 cm s^−1^). The antenna was placed 0.5 cm from the outlet of this tube.

The antenna was cut at the base and inserted (one or two segments) into the recording glass electrode filled with Beadle–Ephrussi Ringer and connected to a pre-amplifier probe connected to a high impedance DC amplifier interface box (IDAC-2; Syntech), while the reference electrode was connected to the antennal tip, after cutting the distal segment. Chromatograms and EAGs from runs with lilac, using unmated females (*n* = 6) and cotton headspace, using mated females (*n* = 6), were superimposed and averaged.

For EAG recordings, odour stimuli were produced by loading filter papers (1 × 1 cm) with solutions of test compounds (10 µl) and inserting them into the glass Pasteur pipettes. Dilutions of compounds were prepared in redistilled *n*-hexane, except for benzyl alcohol, which was dissolved in diethyl ether. Pipettes with formulated filter papers were kept during 30 min in a fume hood to allow for solvent evaporation.

The pipette was connected via a silicone tube to a stimulus generator (CS-55 stimulus controller; Syntech Kirchzarten, Germany) and the tip of the pipette was inserted into the glass tube with an air-flow directed towards the antenna. Stimuli were produced by puffing air through the pipette during 0.5 s. Each set of odour stimuli was tested on one antenna (*n* = 10). Hexane was used as a solvent blank, as the first and last stimulus for every replicate. EAG responses were normalized according to a standard stimulus (1 µg α-humulene), after every two test stimuli.

### (g) Three-dimensional surface map of the antennal lobe

Moth heads were fixed in 4 per cent paraformaldehyde in 0.01 M phosphate-buffered saline containing 0.25 per cent Triton X-100 phosphate buffered saline with the detergent Tween (PBST) and kept overnight at 4°C. Brains were dissected out of the head capsules, washed for 4 × 10 min at room temperature in PBST and preincubated with 2 per cent normal goat serum (NGS) in PBST overnight at 4°C. Brains were then incubated for 3 days at 4°C in 3 per cent anti-synapsin (Hybridoma, University of Iowa, IA, USA), 3 per cent Alexa Fluor 546 phalloidin (Invitrogen) and 2 per cent NGS dissolved in PBST. They were washed in PBST for 3 × 10 min at room temperature and then incubated for 3 days at 4°C in 2 per cent anti-mouse Alexa Fluor 488 (Invitrogen) with 2 per cent NGS in PBST. After washing in PBST for 3 × 10 min at room temperature, they were mounted on glass slides, using spacer rings (Secure-Seal imaging spacer, Sigma-Aldrich), in Elvanol.

Brain whole mounts were examined with a laser scanning confocal microscope (Zeiss LSM 510, Carl Zeiss, Jena, Germany) with a 40 × 1.4 oil-immersion differential interference contrast objective. Structures labelled with Alexa Fluor 488 were excited using an Argon laser at 488 nm and detected using a 505 nm long-pass filter, whereas Alexa Fluor 546-labelled structures were excited with a HeNe laser and detected using a 560 nm long-pass filter. Stacks of X–Y confocal images were scanned with a step size of 1 µm and the images were stored at a size of 1024 × 1024 pixels at 12 bit colour depth.

Images were analysed using Amira v. 3.0 software (Visage Imaging, Berlin, Germany). Left and right antennal lobes (ALs) from 14 preparations were mapped; each glomerulus was manually labelled, reconstructed and numbered from the most anterior to the most posterior. Glomeruli of different individuals were compared and matched.

### (h) Calcium imaging

Moths were fixed in 500 µl plastic pipette tips with the head protruding. Scales were removed and the head was secured with Dental wax (Surgident, Heraeus Kulzer Inc., NY, USA). Part of the cuticula between the compound eyes and the underlying muscles, tracheae and connective tissue was removed to expose the brain. Furthermore, antennal muscles and the oesophagus were severed to minimize movement interfering with recordings. The neurolemma was left complete and great care was taken not to damage the antennal nerves [13].

The animal was placed in a custom-made Plexiglas recording holder and the brain was covered with Calcium-green-2-AM dissolved in 20 per cent Pluronic F-127 in dimethyl sulfoxide (Molecular Probes, Eugene, OR, USA) that was diluted in Beadle–Ephrussi Ringer by sonication for a final concentration of approximately 30 µM. The preparation was kept on ice, in the dark during 1 h.

After rinsing in fresh Ringer, the preparation was placed under the recording microscope (Olympus, Tokyo, Japan) and recordings of 40 frames (4 fps, 200 ms exposure time) were made by an air-cooled CCD camera (TILL Photonics, Gräfling, Germany) at 475 nm excitation, through a 20× (NA 0.50, Olympus) water submission objective. Filter settings were dichroic (500 nm, emission LP 515 nm). Initialization of protocols and analysis of data was performed using Tillvision (TILL Photonics). Odour stimulation started at frame 12 and lasted for 1 s.

Odour responses were analysed by subtracting a solvent recording from the odour recording and normalizing the odour response to a positive control (α-humulene). Background fluorescence (*F*) was defined as the average signal in frames 3–10, before odour stimulation. The relative change in fluorescence (*ΔF*/*F*) was calculated by subtracting *F* from subsequent frames (*F_i_*) and relating the resulting change in fluorescence to the background fluorescence (*F_i_* − *F*)*/F*.

### (i) Wind tunnel bioassay

Behavioural experiments were performed in a wind tunnel made of Plexiglas with a flight section of 180 × 90 × 60 cm, illuminated from above and the side at 2–3 lux. The wind speed was 30 cm s^−1^, incoming air (22–24°C, 50–60% RH) was filtered with active charcoal.

Insects were kept individually, in 2.5 × 12.5 cm glass tubes closed with gauze, in the wind tunnel room for 1 h before testing. Three days-old mated and unmated females were tested individually, in batches of 10 (*n* = 5) with each stimulus. Experiments were performed 1–4 h after onset of the scotophase.

A cotton plant or a cluster of lilac flowers was placed at the upwind end of the wind tunnel. Moths were placed, in glass tubes, on a platform at the downwind end of the tunnel and observed for 5 min. The following steps of the behavioural sequence were recorded: activation (walking and wing-fanning), take-off (flight initiation), upwind flight directed towards the odour source over 75 and 150 cm, and landing at the source. Control tests were performed in an empty tunnel and with a green dummy plant made of polyethylene, which had the same height as the cotton plants.

### (j) Statistical analysis

Fisher's exact probability test was performed to compare the effect of physiological state on the behaviour of *S. littoralis* in the presence of two odour stimuli. Repeated-measures (mixed model) ANOVA was used to compare AL activity in unmated and mated female moths to different dilutions of natural headspace standard extracts and synthetic compounds. Statistical analysis was calculated with GraphPad Prism v. 5.0a.

## 3. Results and discussion

### (a) Post-mating behavioural switch and modulation of the olfactory response

**Figure 1.**
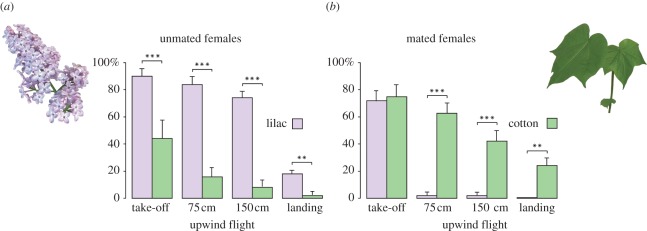
Attraction of (*a*) unmated and (*b*) mated cotton leafworm *Spodoptera littoralis* females in a flight tunnel, towards lilac flowers *Syringa vulgaris* and cotton foliage *Gossypium hirsutum* (mean ± s.e.m.; *n* = 50). Differences between unmated and mated females, for all steps of the behavioural sequence are significant at **p < 0.001 and ***p < 0.0001, according to Fisher's exact probability test.

Females are highly attracted to nectar-rich lilac flowers *Syringa vulgaris* when unmated, whereas mated females instead respond to green foliage of the larval host cotton, *Gossypium hirsutum* (figure 1). Unmated females were activated from rest by both lilac and cotton, but only lilac elicited significant attraction (figure 1). Neither unmated nor mated females responded to unscented dummy plants.

**Figure 2.**
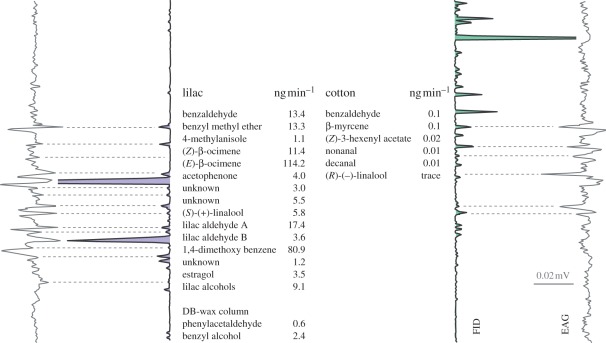
Antennal response of cotton leafworm females to lilac and cotton volatiles. Averaged GC–EAD signal from unmated and mated *Spodoptera littoralis* female antennae to compounds from lilac flower *Syringa vulgaris* (*n* = 6) and cotton foliage *Gossypium hirsutum* headspace (*n* = 6), eluting from a HP-5 capillary column. Compounds eliciting an antennal response are listed in elution order, release rates (ng min^−1^) show emission from a lilac flower cluster and a cotton plant, respectively. Two compounds in the lilac headspace did not separate on the HP-5 column, they were analysed using a DB-wax column (*n* = 7). FID, flame ionization detector; EAG, electroantennogram.

The compounds in lilac and cotton headspace eliciting an antennal response in unmated and mated females, respectively, were identified by GC–EAD and GC–MS, showing 17 active compounds in lilac and six in cotton, with benzaldehyde as the only co-occurring compound (figure 2). The overall volatile release rate was 279.0 ± 86 ng min^−1^ from lilac and 0.1 ± 0.04 ng min^−1^ from cotton. (*E*)-β-Ocimene and β-myrcene were the most abundant compounds in lilac and cotton, respectively. Lilac flowers released (*S*)-(+)-linalool, whereas cotton foliage released trace amounts of (*R*)-(−)-linalool (figure 2) [14,15].

**Figure 3.**
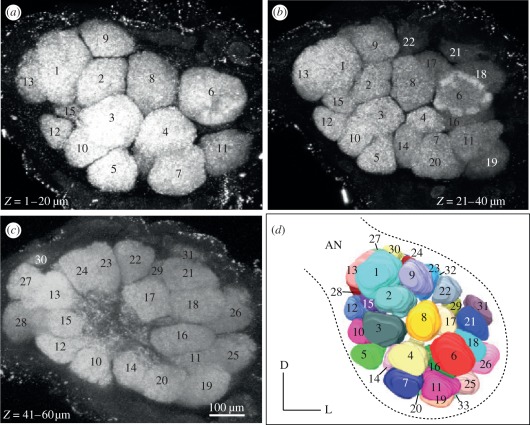
Surface map of the female antennal lobe (AL) of cotton leafworm *Spodoptera littoralis*. Confocal sections, with glomeruli numbered from anterior to posterior (*a*–*c*). Three-dimensional reconstruction of the AL glomeruli that are visible during optical imaging (*d*). Antennal nerve (AN), dorsal (D), lateral (L).

A glomerular map of the anterior surface of the female AL of *S. littoralis* is shown in figure 3. In 14 females, between 30 and 35 spheroid glomeruli were found, comprising all glomeruli that are visible during calcium imaging. Glomeruli 1–4, 6, 8 and 9 were rather consistent in position, shape and size, they were used as landmarks to identify other glomeruli that showed substantial variation between individual females.

**Figure 4.**
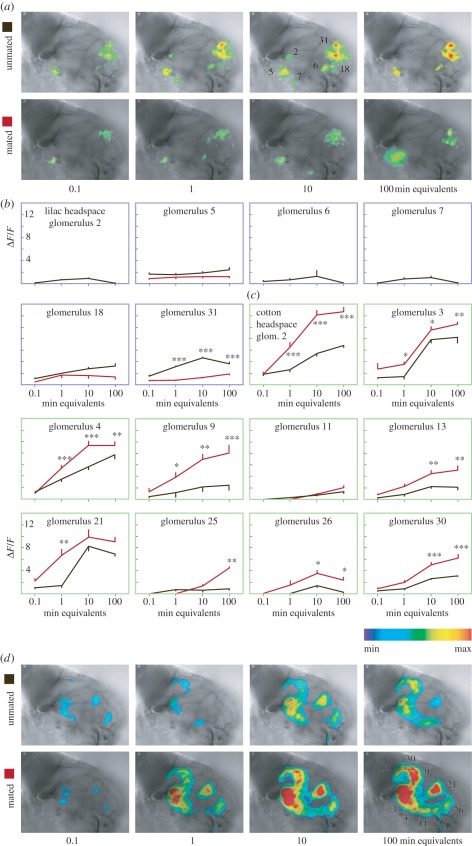
Calcium response of AL glomeruli to authentic lilac and cotton volatile blends. Glomerular activity in the antennal lobe (AL) of unmated (black) and mated (red) cotton leafworm females *Spodoptera littoralis* in response to headspace of lilac flowers *Syringa vulgaris* (*a*,*b*) and cotton foliage *Gossypium hirsutum* (*c*,*d*). False colour-coded images of calcium responses (*a*,*d*) and intensities in relative change of fluorescence (*ΔF/F*) (*b*,*c*), obtained with increasing amounts of headspace, corresponding to the amount of volatiles released during 0.1–100 min from lilac flowers and cotton plants used in behavioural experiments (*p < 0.05, **p < 0.01, ***p < 0.001, two-way repeated-measures ANOVA, mean ± s.e.m., *n* = 5 females).

The response of glomeruli in the ALs of unmated and mated females to lilac flower and cotton foliage headspace was investigated by calcium imaging. Lilac odour produced a stronger response in the AL of unmated than in mated females (figure 4*a,b*), whereas cotton leaf odour had a stronger effect in mated females (figure 4*c*,*d*). The reverse AL glomerular activation pattern by floral and green odour (figure 4) matches the attraction behaviour of unmated and mated females in the wind tunnel (figure 1).

**Figure 5.**
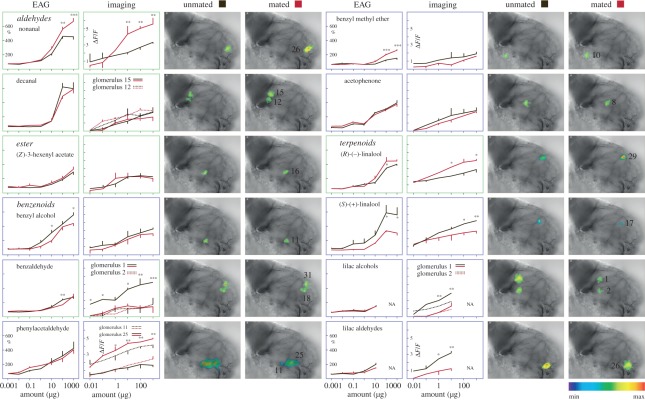
Calcium response of AL glomeruli (false colour-coded images) and antennal (EAG) response, from unmated and mated cotton leafworm females *S. littoralis*, to synthetic lilac and cotton volatiles (*p < 0.05, **p < 0.01, ***p < 0.001, two-way repeated-measures ANOVA, mean ± s.e.m., *n* = 5 females for AL recordings, *n* = 10 females for EAG recordings). Lilac alcohols and aldehydes were not available for tests with amounts higher than 1 µg.

In unmated females, six glomeruli (2, 5, 6, 7, 18 and 31; figures 3 and 4) showed a consistent calcium response to lilac headspace in amounts corresponding to a 1–10 min release from live flowers. Three glomeruli (5, 18 and 31) were consistently activated also in mated females (figure 4*a*,*b*). Synthetic standards of four GC–EAD-active lilac compounds (benzaldehyde, (*S*)-(+)-linalool, lilac alcohols and lilac aldehydes; figure 2) elicited a significantly stronger glomerular response in unmated, compared with mated females, while phenylacetaldehyde elicited a stronger antennal response in mated females (figure 5).

In the AL of mated females, cotton headspace elicited a strong response (figure 4), despite the much smaller amounts of volatiles released from cotton foliage than from lilac flowers (figure 2). Nine of 10 activated glomeruli produced a significantly stronger calcium signal in mated, compared with unmated females (figure 4). Two standards of GC–EAD-active cotton compounds, nonanal and (*R*)-(−)-linalool, gave a significantly stronger antennal and glomerular response in mated females (figure 5). The two enantiomers, (*R*)-(−)- and (*S*)-(+)-linalool, were found to activate the neighbouring glomeruli 17 and 29 (figures 3 and 5). Interestingly, the activation pattern of these two glomeruli and the peripheral response was inverted after mating (figure 5) and this is paralleled by the respective occurrence of the linalool enantiomers in lilac and cotton (figure 2). In the hawk moth *Manduca sexta*, where (*S*)-(+)-linalool is expressed in a female-specific AL glomerulus, females show an enantioselective egg-laying response: they oviposit more on host plants emitting (*S*)-(+)-linalool than on plants emitting (*R*)-(−)-linalool [16].

### (b) Encoding of chemical signals in odour-specific ensembles of glomeruli

Lilac and cotton odours are encoded in different sets of glomeruli in the AL, because they emit different chemical signals. Mating downregulates the activation of the ensemble of glomeruli responsive to lilac odour, and upregulates in parallel the ensemble of glomeruli tuned to cotton odour (figure 4). Not all glomeruli activated by headspace could, however, be associated with individual chemicals, and vice versa, several glomeruli responding to single compounds were not activated by blends (figures 4 and 5). Calcium activity in the AL reflects input by afferent olfactory receptor neurons as well as interglomerular communication by lateral connections through interneurons [17], which probably accounts for the observed discrepancies between activation by individual compounds and the entire headspace blend. In the Oriental fruit moth *Grapholita molesta*, a new glomerulus type was found to respond synergistically to a blend of plant volatiles, corresponding to the behavioural synergism obtained with this volatile blend [18].

A 1 min release equivalent of cotton headspace is approximately 3.5 orders of magnitude more dilute than lilac flower headspace. A conspicuous AL response, together with a strong upregulation after mating, underlines the significance of this faint green-leaf oviposition signal: cotton leafworm females search for larval host plants soon after mating. By contrast, lilac headspace produces a comparatively sparse glomerular representation (figures 2 and 4). This may be an adaptation to the abundant release of volatiles from flowers or it may even reflect the behavioural hierarchy of these signals—food intake is not required for mating or egg-laying.

The cotton leafworm, as other noctuid moths, is attracted to a range of plants [19], but the females are not indiscriminate and prefer, for example, cotton over cabbage for oviposition [20]. Many of the antennal active lilac and cotton volatiles (figure 2) are ubiquitous compounds found in other broad-leaf plants, but the attractant blends may nonetheless contain a backbone of essential compounds, in addition to redundant, interchangeable compounds, that signal suitable oviposition substrates [21,22]. A topic for future research is accordingly which compound blends essentially encode floral and green odour, and whether these compounds route to particular topographic regions or glomeruli of the AL.

### (c) Mechanisms of odour modulation

This leads to the question of how modulation in glomerulus clusters associated with lilac and green odour is orchestrated. Figure 4 indicates that these two olfactory channels may be selectively regulated. The modulation of AL networks involves several factors, including biogenic amines, neuropeptides and gonadotrophic hormones, i.e. juvenile hormones and ecdysteroids [4,23]. Local interneurons have been shown to mediate gain control in the olfactory circuit through presynaptic interactions with olfactory receptor neurons [24–26]. Considering their diverse neurotransmitter profiles, morphology and physiology, this type of neuron would be suitable to modulate select glomeruli ensembles [27,28]. In *D. melanogaster*, starvation modulates the olfactory representation of food odour in specific glomeruli in the AL: the global signal insulin regulates presynaptic activity in odorant receptor neurons dedicated to food odours via expression of a local neuropeptide [29]. Differences in the antennal response of unmated and mated females (figure 5) suggest that peripheral modulation may be a contributing factor. Likewise, the downregulation of olfactory receptors in response to blood feeding has been demonstrated in mosquitoes [30].

### (d) Plasticity of the insect olfactory system and chemical ecology

Mating induces profound physiological changes, especially in females, that necessitate changes in foraging and reproductive behaviour. A first account of the consequences of mating in an insect herbivore concerns the Mediterranean fruitfly*, Ceratitis capitata*. The females become attracted to host fruit instead of male pheromone following mating [6]. Moths are known to be attracted to flowers and their larval host plants, for example, cotton bollworm *Helicoverpa armigera* [31,32], cabbage looper *Trichoplusia ni* [33,34] and grapevine moth [22,35]. However, a modulation of the olfactory system, which accounts for post-mating behavioural changes, has been studied only in males of the noctuid moth *A. ipsilon*, which undergo a transient response inhibition to female sex pheromone [10].

In cotton leafworm females *S. littoralis*, mating leads to a shift in olfactory intensity coding of plant odours. A clear-cut modulation of odour-driven behaviour as a function of mating is a useful substrate for studies of the neural mechanisms underlying state-dependent choice. Furthermore, the behavioural application of odour templates in decisions to select plants for feeding and oviposition is central for plant–insect interactions and speciation events in insect herbivores [36–38]. Studies of neural and behavioural mechanisms that tune the olfactory system to odour blends contribute to resolve the question of how insect herbivores assign value to sensory information from plants and how they choose their plant hosts. Studies of olfactory modulation and changes in value-based decisions contribute to the question of how insects acquire new odour templates of ecological relevance, and how new hosts and food sources are acquired.
